# The Impetus of Artificial Intelligence on Periodontal Diagnosis: A Brief Synopsis

**DOI:** 10.7759/cureus.43583

**Published:** 2023-08-16

**Authors:** Priyanka Cholan, Lakshmi Ramachandran, Santo G Umesh, Sucharitha P, Anupama Tadepalli

**Affiliations:** 1 Periodontics, Sri Ramaswamy Memorial (SRM) Dental College & Hospital, Chennai, IND; 2 Periodontics & Oral Implantology, Sri Ramaswamy Memorial (SRM) Dental College & Hospital, Chennai, IND; 3 Periodontics, Sri Ramaswamy Memorial (SRM) Dental College, Chennai, IND

**Keywords:** oral health care, art of diagnosis, diagnosis, maxillofacial abnormalities, periodontal diseases, deep learning artificial intelligence

## Abstract

The current advances in digitized data additions, machine learning and computing framework, lead to the swiftly emerging concept of “Artificial Intelligence” (AI), that are developing into areas that were formerly contemplated for human expertise. AI is a relatively rapid paced mechanics wherein the computer technology is tuned to perform human tasks. An auxiliary domain of AI is machine learning (ML), and Deep learning, a subclass of ML technique comprehends multi-layer mathematical operations. AI-based applications have tremendous potential to improve and systematize patient care thereby alleviating dentists from laborious regular tasks, and facilitate personalized, predictive and preventive dentistry. In the dental clinic, AI can execute a variety of easy tasks with greater accuracy, minimal manpower, and with fewer mistakes over human equivalents. These tasks range from appointment scheduling and coordination to helping with clinical evaluation and therapy. Besides, this could assist in the early diagnosis of dental and maxillofacial abnormalities like periodontal ailments, root caries, bony lesions, and facial malformations in addition to automatically identifying and classifying dental restorations on digital radiographs. This brusque narrative review describes the AI-based systems, their respective applications in periodontal diagnosis, the multifarious studies, possible limitations and the predictable future of AI-based dental diagnostics and treatment planning.

## Introduction and background

Scientists and researchers have always been intrigued by the brain, yet the scientific community has never truly grasped how to build a perfect simulation of the human brain [1,2]. The development of “artificial intelligence” (AI) has been the focus of intense scientific research for many decades now, but the first mention of the discipline of applied computer science currently known as artificial intelligence was made by John McCarthy in 1956 [3]. AI, a method that utilises computer technology and machines to simulate human-like reasoning, judgement, and intelligent conduct, is frequently referred to as the fourth industrial revolution.

It is an outstretched field that focuses on developing intelligent and resourceful machines that can carry out tasks that conventionally require human intellect. Despite the fact that it's an interdisciplinary science with several new perspectives, progressions both in the field of machine learning and deep learning are ushering as a brand-new era in almost every vicinity of the engineering and medical arena. This groundbreaking revolution in dentistry spurted with digitization of dental records and utilized dental records to automatically locate anatomical landmarks, identify diseases, and categorize diseases. Artificial intelligence has bolstered a fast fleet providing an infinite array of potential applications in all aspects of dentistry. Currently, experts focus on smart tools because of the advancement of medical knowledge and the complexity of decisions relating to diagnosis and treatment. By implementing these tools and processes, medical professionals can remarkably reduce the likelihood of making inaccuracies brought on by fatigue or a lack of experience with disease diagnosis and treatment. Additionally, we could analyse the medical database more thoroughly and precisely by employing AI-based tools. Therefore, we need models with lower errors and more reliability for this purpose.

## Review

The fundamental nomenclature required to comprehend the artificial intelligence conceptual framework are:

Artificial intelligence is the term used to describe the idea that machines are capable of operating in a manner that duplicates or mimics human intelligence. Different elements of AI, including human-like communication or decision-making, are possible.

Chatbot is a computer program that simulates human-to-human interaction by interacting with users through text or voice commands.

Cognitive computing is a precisely described term for artificial intelligence. Some organizations' marketing departments use it to get over the sci-fi stigma that occasionally accompanies AI.

Deep learning is an AI feature that mimics the way the human brain works by learning from the structure of data rather than from an algorithm that is programmed to perform a certain task.

Machine learning is a branch of AI that is especially concerned with creating algorithms that will enable computers to pick up new information and adapt on their own, without the aid of a human.

A neural network is a type of computer program created to mimic how the human brain works. Existing neural networks are capable of performing a variety of tasks involving voice, vision, and board game strategy, even though scientists are still working on building a machine model of the human brain.

The Turing test examines an artificial intelligence's capacity to imitate human behavior and language. The machine succeeds after being evaluated by a person if its output is identical to that of the participant.

Variance is the degree to which a machine-learning model deviates from its intended function while it is being taught. Models with large variance are adaptable, but because they depend on their training data, they are prone to overfitting and have low prediction accuracy [4].

Contrasting featuresof AI

Natural intelligence strives to adapt to new circumstances by melding together numerous cognitive processes, in juxtaposition to artificial intelligence. The biological brain system and its capacity for learning through repetition serve as an inspiration for artificial neural networks [5]. Artificial intelligence-based mathematical models are now used to support some diagnostics, and neural networks possess the ability to assimilate in order to make a diagnosis from the data provided to it. Although the neural networks initially appear to be complex, it is simple to integrate them into the healthcare industry [6]. Table 1 depicts the salient features of AI and Natural Intelligence.

**Table 1:** Salient features of artificial intelligence

S.NO	PARAMETERS	NATURAL INTELLIGENCE	ARTIFICIAL INTELLIGENCE
1.	PRINCIPLE	Combining a variety of cognitive processes is the goal of human intelligence in order to adjust to changing circumstances.	AI aims to develop machines that can behave like people and carry out tasks that would typically be done by people.
2.	PROGRESSION	According to the theory of natural intelligence, humans are born with the capacity to reason, think critically, and perform other cognitive tasks.	AI was created by humans and has much greater cognitive abilities than natural human beings.
3.	PERFORMANCE	People make use of their brains' memory, processing speed, and cognitive skills.	AI-powered gadgets need to process commands and data in order to work
4.	MODE OF OPERATION	When it comes to speed, humans cannot match machines or artificial intelligence.	Compared to individuals, computers are capable of rapid processing of data
5.	LEARNING CAPABILITY	The foundation of human intelligence is developed through the process of learning from a variety of situations and experiences.	They can only learn things by being exposed to them and practising them repeatedly; they can never develop a thought process that is exclusively human.
6.	DECISION MAKING	Human decision-making may be influenced by subjective factors that are not solely based on data. Because it evaluates using all available information.	When it comes to making decisions, AI is incredibly impartial.
7.	PRECISION	When it comes to human insights, there is almost always a chance for "human error," which refers to the possibility that some subtleties may be missed occasionally.	Because AI is based on a set of guidelines that can be updated, it can consistently produce accurate results.
8.	MODIFICATIONS	The human mind is able to change its viewpoints in response to the shifting circumstances of its environment. People can remember information as a result, and they also perform well in a variety of activities.	Artificial intelligence requires considerably more time to adjust to unneeded changes.
9.	FLACCIDITY	Juggling several tasks at once demonstrates that multitasking requires the capacity to use good judgement. Similar to how a framework can pick up tasks one at a time.	Only a small portion of the tasks can be completed simultaneously by artificial intelligence.
10.	INTER- NETWORKING	In terms of their capacity for conceptual assimilation, degree of self-awareness, and receptivity to other people's emotions, humans outperform other social animals. This is due to the social nature of people.	The ability to recognise related social and enthusiastic indicators is still a skill that artificial intelligence is still working on.
11.	APPLICATION	It could be characterized as creative or inventive.	Since robots are unable to think in the same ways that people do, it is not possible for it to be creative or inventive.

Designing of neural networks

**Figure 1:**
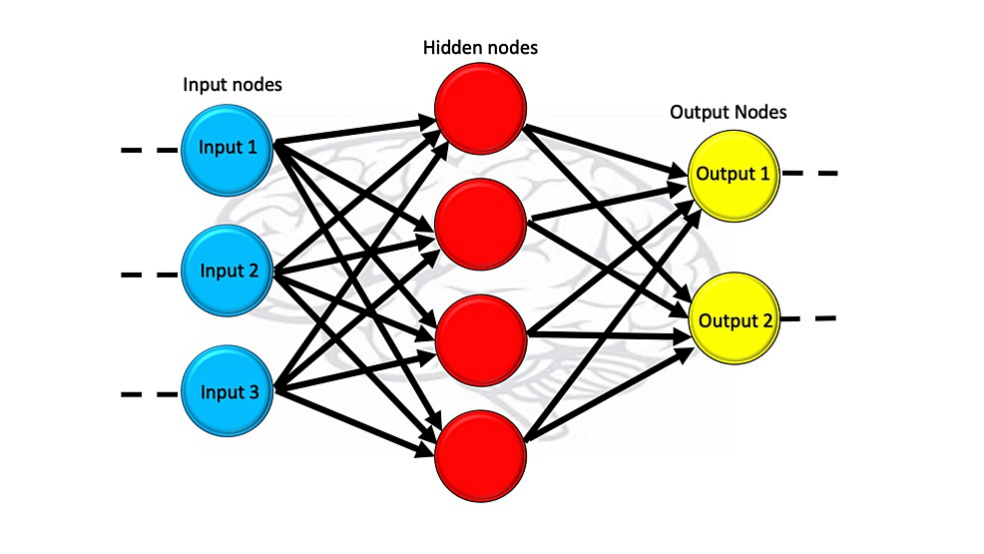
The image depicts how the artificial network works

Artificial Neural Networks (ANN) is essentially a feed-forward network because of the way information travels through it from one layer to another without touching a node twice. This type of neural system patterned around neurons works similarly to a human brain, recognizes patterns in raw data, and helps solve complex processes [7]. The ANN mimics the brain by upgrading itself with every new input it receives and makes independent improvement or is continuously improving. The ANN is based on many integrated layers of nodes and all layers are in charge of inputting, developing, and outputting data to the deeper layers as illustrated in Figure 1 [7]. The uniqueness of this highly interlayered neural network system helps in understanding and learning complex things. ANNs employ distributed information storage throughout the network, contrasting with traditional programming's reliance on specific databases. This characteristic contributes to the system's fault tolerance.

Moving on to Convolutional Neural Networks (CNNs), they deserve recognition for their role in image and video recognition, recommendation systems, as well as image analysis and classification. Prior to their introduction, manual labor was required for object identification in images. However, CNN has revolutionised the process by leveraging the principles of linear algebra to uncover patterns in images. Additionally, it possesses the ability to automatically detect features without the need for human supervision, giving it distinct advantages over its predecessors. Nevertheless, there are challenges with utilising CNNs for effectively handling variations in presented data. Conversely, CNNs face difficulties when processing images containing partially hidden objects and classifying rotated or tilted images as it's unable to encode an object's orientation and position, limiting its ability to process spatially invariant data. It is worth noting that training CNN necessitates the use of multiple graphical units (GPU), which contributes to its high computational demands [7].

**Figure 2:**
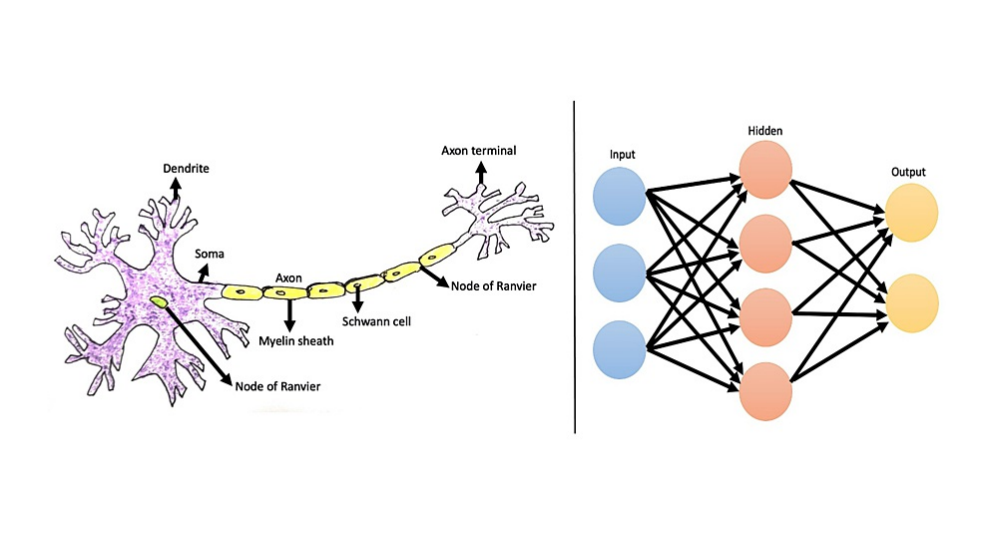
Perception of neural network The image illustrates the features of neuron, unit of neural network in contrast to the Artificial Neural Networks (ANN).

Recurrent Neural Networks (RNNs) possess a distinctive capability to process historical and present data, as well as retain information, thereby overcoming the limitations of feed-forward networks. RNNs offer unique advantages compared to other types of Neural Networks, which open up a wide range of possibilities for users, but they also present certain challenges. The potential benefits of RNNs include: (1) They are the sole Neural Networks equipped with memory and dual data processing, enabling the mapping of multiple inputs and outputs. (2) Unlike other algorithms that provide a single output for a given input, RNNs can handle mappings of many-to-many, one-to-many, and many-to-one input-output relationships. (3) Most notably, RNNs exhibit a profound understanding of sequences and their contextual relationships, distinguishing them from other Neural Networks (Figure 2). However, along with these benefits, there are a few challenges to consider, such as the slow, time-consuming, complex nature of RNN training, making implementation complex and challenging [7]. Table 2 describes the distinguishing features of ANN, CNN and RNN.

**Table 2:** Prime features of ANN, CNN and RNN ANN: Artificial Neural Networks; CNN: Convolutional Neural Networks; RNN: Recurrent Neural Networks

	ANN	CNN	RNN
BASICS	Simplest	Popular	Advanced and complex
STRUCTURAL LAYOUT	Information flows in one direction	Information flows through more convolutional layers	Information flows in different directions
DATA TYPE	Tabular and text data	Image data	Sequence data
COMPLEXITY	Simple in contrast	More powerful	More powerful
COMMENDABLE FEATURE	Ability to work with incomplete knowledge	Accurate in recognizing images	Memory and self-learning
SPATIAL RECOGNITION	no	yes	no
RECURRENT CONNECTIONS	no	no	yes
MAIN DRAWBACK	Hardware dependence	Large training data required	Slow and complex training
USES	Complex problem solving	Image recognition	Natural language processing and sentimental analysis

Artificial intelligence in periodontal diagnosis

Periodontal disease is a chronic inflammatory condition triggered by a dysbiotic microflora in the gingival sulcus. This imbalance activates the innate immune response through various receptors found in host cells, leading to intra- and inter-cellular signalling. Consequently, the host's immuno-inflammatory response is initiated, influenced by multiple factors that contribute to both the cause and modification of the disease. These factors act simultaneously and interactively. The disease manifests as a localized microbial burden, provoking an inflammatory reaction and tissue deterioration and is characterized by alternating periods of exacerbation and remission.

Over the past 10 years, the usage of CNNs in periodontal and dental research has increased steadily. The number of articles utilising CNNs has exponentially increased since the first ones were published in the early 2010s. Over 2000 studies featuring CNN in the title were registered on PubMed in 2021. This tool has limitless potential and continues to advance along with the designs, offering both general usefulness and task-specific utility. However, the bulk of studies used pixel-by-pixel annotation methods with few of them switching over to the “Dye Staining” or “Grab Cut” methodology. The profitable nature of these tools in a “real world setting” won't be established until studies comparing performance against truly independent dentist performance in a clinical situation are presented or until literature offering baseline information on the efficacy of examiners is generally recognised [8].

In contrast to evidence-based dentistry (EBD), machine learning (ML) lacks a system to monitor the quality of input medical data and assess the level of bias. EBD demonstrates a more comprehensive understanding, making decisions based on multiple data sources to minimize bias. Due to these limitations, some clinicians have reservations about ML due to its “black box” mechanism, which makes it challenging to explain how specific results are obtained. ML represents a novel approach in the medical field, aiming to enhance diagnosis and predict treatment outcomes by identifying patterns and associations within medical datasets. Indeed, while current ML applications predominantly rely on uniform datasets, ML has the potential to incorporate information from EBD, which utilizes diverse data types for diagnosis. By leveraging ML, EBD can discover meaningful connections between medical data and diseases, leading to improved and personalized diagnoses. EBD and ML complement each other, offering clinicians a comprehensive toolkit to enhance their medical practice. By utilizing both EBD and ML, clinicians can maximize their advantages and make more informed decisions in patient care [9].

AI and current diagnostic modalities

The diagnostic approach for periodontal disease can be complex and time-consuming. Currently, it relies on invasive radiographic tests that lack clear visualization of soft tissue or manual periodontal probing, which is subjective and dependent on the clinician's expertise [10]. However, incorporating microbiological studies and periodontal biomarker analysis can enhance clinical diagnosis. Recent technological advancements have made periodontal tissue imaging possible, playing a crucial role in case diagnosis. Optical coherence tomography and periodontal ultrasonography have been used in previous research to demonstrate the effectiveness of non-invasive periodontal scanning. However, these methods are time-consuming and still require expertise in the interpretation of ultrasonography images of periodontal tissue, as it can be difficult for periodontists.

The application of Artificial Intelligence (AI) in interpreting generated images holds promise in overcoming these challenges. CNNs can automatically analyze X-ray data and aid in diagnosing periodontal disease. This can help to address the limitations of radiography, such as radiation exposure and difficulties in observing soft tissue, which further hinder the monitoring of disease progression and treatment outcomes [11-16].

Potential applications of AI in periodontal diagnosis

Disease Classification

Periodontitis, a prevalent inflammatory disease in humans, is primarily caused by prolonged bacterial infection. Research focusing on the pathobiology of periodontal disease helps expand our understanding of this condition [17]. In 2014, Ozden et al. developed a classification system utilizing support vector machine (SVM), decision tree (DT), and ANNs to identify different types of periodontal diseases. The study involved 150 patients divided into two groups: a training group consisting of 100 patients and a testing group consisting of 50 patients [18]. Risk factor codes, periodontal data, and radiographically measured bone loss were used as inputs for the classification system, which produced six distinct periodontal conditions as outputs. The accuracy of the proposed methods was evaluated based on their resolution and processing time.

Based on clinical research conducted on the 150 patients, it was found that DT and SVM performed exceptionally well in accurately classifying periodontal diseases. The SVM achieved a 98% accuracy with a total computation time of 7.00 seconds, while the DT achieved the same accuracy with a slightly longer computation time of 19.91 seconds. On the other hand, ANN showed a lower performance with an estimated accuracy of 46%. The researchers concluded that SVM and DT were both comprehensible and practical decision-making tools for predicting periodontal disease, as they were simple enough to be understood while encompassing the various factors associated with periodontal destruction.

Prognosis and Risk Assessment

Neural networks can be an effective tool for enhancing medical behaviour and maximising the benefit from treatment expenditures, as demonstrated by Amiri et al. in 2006. In the healthcare industry, the use of AI for prognosis and risk assessment has been regularly studied [19]. Levenberg-Marquardt algorithm-trained natural networks have been successfully applied in periodontal disease risk assessment, according to Shankarapillai et al. [20]. In 2012, Moghimi et al. used a genetic algorithm and artificial neural network to predict the size of hidden canines and premolars. The results demonstrated that the proposed method was more accurate and effective than regression analysis at predicting the size of hidden canines and premolars [21]. According to earlier studies, artificial neural networks' distinctive capacity to recognise differences, classify, and identify diseases is potentially effective and significant.

Disease Detection

From dental plaque microscopy pictures, Aberin and Goma employed a CNN system to identify periodontal disorders. To be more precise, they employed the CNN method to match photos of individuals with healthy and unhealthy periodontium to the corresponding conditions, and they were 75.5% accurate [22]. Comparably, Balaei et al. [23] used a CNN system to identify periodontal disease from intraoral pictures and attained an accuracy of 66.7% for disease detection and 91.6% for pretreatment evaluation. Both these investigations demonstrate that CNN systems can be utilised effectively to ascertain periodontal health.

From 1044 periapical radiography pictures, Lee et al. employed a CNN method to identify periodontally hazardous teeth, classifying the teeth as healthy, moderate, and severe [4]. The lowest and best accuracy were calculated independently for the mandible and maxilla, and they came out with 73.4% and 82.8%, respectively. When it came to accurately diagnosing teeth with periodontal insufficiency, they said that their CNN technique appeared to hold promise. Given that their study included clinical assessments, it may be regarded as being more precise [24]. Using their CNN approach, Krois et al. assessed radiographic pictures from 2001 and reported sensitivity, specificity, and accuracy values of 0.81, 0.81, and 0.81, respectively [25]. They also documented and statistically compared the evaluation outcomes from six dentists.

In a manner similar to this, Kim et al. reported that this approach may lessen their obligations of dental radiologists who interpret images by successfully evaluating periodontal bone loss from panoramic radiographs using a CNN system [26]. When utilising a CNN system to assess bone loss and periodontitis staging in accordance with the standards of the “2017 World Workshop on the classification of periodontal and peri-implant diseases and conditions,” Chang et al. reported high accuracy and reliability [27-30]. The studies on the application of AI in periodontal diagnosis are enlisted in Table 3.

**Table 3:** Comprehensive literature review - AI in periodontal diagnosis COPD: Chronic obstructive pulmonary disease; NHANES: National Health and Nutrition Examination Survey III; CNN: Convolutional neural network; MLP: Multilayer perceptron; RBNN: Radial basis function neural network; GAN: Generative adversarial networks; IoU: Intersection over Union; PCT: Periodontally compromised teeth.

S.NO	AUTHOR AND YEAR	TITLE	TYPE OF NEURAL NETWORK USED	INFERENCE
1.	Vollmer et al., 2022 [10]	Associations between Periodontitis and COPD: An Artificial Intelligence-Based Analysis of NHANES III	CNN, MLP, RBNN	Deep learning and machine learning algorithms can estimate COPD cases using demographic and dental health characteristic factors, according to study results on an extensive population.
2.	Alotaibi et al., 2022 [13]	Artificial intelligence (AI) diagnostic tools: utilizing a convolutional neural network (CNN) to assess periodontal bone level radiographically—a retrospective study	CNN	The deep CNN algorithm (VGG-16) is useful for detecting alveolar bone loss in periapical radiographs and can accurately assess the level of bone loss in teeth. The results imply that machines can work more efficiently based on level classification and characteristics of captured picture analysis. With further optimisation of the periodontal dataset, it is predicted that a computer-aided detection system will emerge as an efficient technique for assisting in the diagnosis and staging of periodontal disease.
3.	Kearney et al., 2022 [31]	A generative adversarial inpainting network to enhance prediction of periodontal clinical attachment level	CNN, GAN	Artificial intelligence was created and employed to forecast clinical attachment level in place of clinical measurements. A generative adversarial inpainting network with partial convolutions was developed, assessed, and validated to predict clinical attachment level. The inpainting technique was found to be superior to non-inpainted techniques and to be within the 1 mm clinician-determined measurement threshold.
4.	Chifor et al., 2022 [14]	Automatic Segmentation of Periodontal Tissue Ultrasound Images with Artificial Intelligence: A Novel Method for Improving Dataset Quality	R-CNN and U-Net convolutional neural network models	Higher IoU following model retraining using the corrected dataset demonstrated the beneficial effects of the suggested quality check and correction technique by measuring the operator's ground truth segmentation in the 3D space.
5.	Piel et al., 2022 [32]	Artificial Intelligence Aiding In The Periodontal Assessment	CNN	CNN can help the clinician read and identify images more quickly, freeing up more appointment time for services like cleaning and patient education.
6.	Xu et al., 2022 [15]	Evaluation of the Effect of Comprehensive Nursing Interventions on Plaque Control in Patients with Periodontal Disease in the Context of Artificial Intelligence	CNN	In order to increase oral health awareness and give an accurate diagnosis of plaque disease, this study built a convolutional neural network-based oral dental disease diagnosis system for oral care interventions. We persistently and permanently insist on comprehensive daily plaque removal in order to improve patients' physical health and quality of life in those with periodontal disease. We accomplish this by urging patients to take appropriate care of their oral health.
7.	Khaleel and Aziz, 2021 [5]	Using Artificial Intelligence Methods For Diagnosis Of Gingivitis Diseases	Principal Component Analysis (PCA) algorithm, Self-Organizing Map (SOM) algorithm and the Fuzzy Self-Organizing Map (FSOM) network algorithm, Bat swarm algorithm	The BAT is the most accurate method in this study because it had a higher diagnosis accuracy for gingivitis disease equal to (97.942%) in the testing state.
8.	Bayrakdar et al., 2020 [11]	Success of artificial intelligence in determining alveolar bone loss from dental panoramic radiography images	CNN	Periodontal bone loss is successfully detected by the CNN system. In the future, oral physicians may use it to make diagnosis and treatment planning easier.
9.	Farhadian et al., 2020 [16]	A decision support system based on support vector machine for diagnosis of periodontal disease	Support vector machine (SVM)	The best performance was demonstrated by the radial kernel function, which had an overall hypervolume under the manifold (HUM) value of 0.912 and an overall correct classification accuracy of 88.7% when used in the design of the SVM classification model. The findings of the current study demonstrate that the created classification model performs reasonably well in predicting periodontitis.
10.	Lee et al., 2018 [4]	Diagnosis and prediction of periodontally compromised teeth using a deep learning-based convolutional neural network algorithm	CNN	The diagnosis and predictability of PCT could be evaluated with the help of the deep CNN algorithm. The diagnosis and predictability of PCT could thus be evaluated using the deep CNN algorithm. As a result, the deep CNN algorithm could be used to assess the diagnosis and predictability of PCT. Therefore, the deep CNN algorithm could be used to assess the diagnosis and predictability of PCT.

Financial dimensions of AI

From the perspective of periodontal diagnosis, AI is currently in its early stages of development and the lack of comparative studies for cost analysis, with and without AI, hinders further assessment. Schwendicke et al. identified early-stage caries utilising AI to enhance the identification in bitewing X-rays and it was proved to be a cost-effective approach [33]. This cost-effectiveness was rooted in its heightened ability to sensitively detect initial caries lesions, enabling their non-restorative management and thereby sidestepping the expensive need for later extensive treatments [34]. The overall application of AI demonstrated the potential to enhance healthcare while mitigating expenses. This is particularly notable due to the substantial and progressively accumulating costs associated with a patient's lifelong care, predominantly attributed to restoration, prosthetic, and periodontal interventions. Nevertheless, it is crucial to acknowledge the dual nature of this situation. AI also carries its own limitations. The foremost concern revolves around the significant initial setup costs. Given that AI systems require constant updates, both in terms of hardware and software, to align with the latest requisites, this incurs ongoing expenses. Uncertainty in the realm of ethical considerations also looms over AI's application, as it involves sensitive medical data used for both training and testing purposes.

## Conclusions

AI is poised to have a significant impact on future healthcare options, particularly in the realm of precision medicine, which is widely recognized as a crucial asset in healthcare. While early attempts at AI-powered recommendations for diagnosis and therapy have posed challenges, it is anticipated that AI will eventually become proficient in this field. Automatic segmentation powered by AI assists operators in expediting analysis, particularly for those not accustomed to visual analysis, enabling clinicians to provide meticulous patient care. Given the rapid advancements in AI for imaging analysis, it is likely that the majority of radiology and pathology images will eventually be reviewed by AI systems. Additionally, speech and text recognition technologies are expected to become more prevalent for patient communication and clinical note transcription.

The biggest hurdle for AI in various healthcare sectors lies not in determining its capabilities but rather in ensuring its acceptance in routine clinical practice. To achieve widespread adoption, AI systems must receive endorsement from regulatory bodies, integration into public healthcare systems, standardization for consistent functioning, guidance and training for clinicians, and adequate funding for continuous operation. While these challenges will eventually be resolved, it will likely take longer than the technological advancements themselves. However, it is increasingly evident that AI technologies cannot completely replace physicians; rather, they will augment healthcare providers in delivering patient care and clinical practitioners may shift towards roles and work schedules that maximize the utilization of unique human abilities such as compassion, motivation, comprehensive assimilation and overall a patient-centric healthcare system.
